# The Evolving Concepts of Cancer Stem Cells in Head and Neck Squamous Cell Carcinoma

**DOI:** 10.1155/2014/842491

**Published:** 2014-01-21

**Authors:** Amit Shah, Shilpa Patel, Jigna Pathak, Niharika Swain, Shwetha Kumar

**Affiliations:** Department of Oral Pathology & Microbiology, M.G.M. Dental College & Hospital, Kamothe, Navi Mumbai 410209, India

## Abstract

There is increasing evidence that the growth and spread of cancers is driven by a small subpopulation of cancer stem cells (CSCs)—the only cells that are capable of long-term self-renewal and generation of the phenotypically diverse tumor cell population. CSCs have been identified and isolated in a variety of human cancers including head and neck squamous cell carcinoma (HNSCC). The concept of cancer stem cells may have profound implications for our understanding of tumor biology and for the design of novel treatments targeted toward these cells. The present review is an attempt to conceptualize the role of CSCs in HNSCC—its implication in tumorigenesis and the possible additional approach in current treatment strategies.

## 1. Introduction

Global increase in incidence and mortality associated with head and neck squamous cell carcinomas (HNSCC) have intensified efforts in the field of research pertaining to tumor biology and therapeutics. HNSCC is one of the most prevalent types of malignancy worldwide. The mortality due to HNSCC is mainly caused by local recurrence and cervical lymph node metastasis and occasionally by distant organ metastasis. Research in cancer therapeutics has helped in targeting pathways that appear to contribute in tumourigenesis and metastasis with greater efficacy and fewer unwanted side effects. An important premise guiding this work is the cancer stem cell hypothesis. The cancer stem cell (CSC) theory of tumourigenesis was originally proposed in the late 1970s and was first described in hematologic malignancies in 1994 [1]. Since then, CSCs have been identified in multiple other solid organ malignancies, including Central Nervous System (CNS), pancreatic, lung, colon, and recently HNSCC [2–6].

The consensus definition of a cancer stem cell that arrived at an “American Association of Cancer Research Workshop on cancer stem cell” is a cell within a tumor that possesses the capacity to self-renew and to cause the heterogeneous lineages of cancer cells that comprise the tumor [7]. Various alternative terms have been used in the literature, such as “tumor-initiating cell” and “tumorigenic cell” to describe putative cancer stem cells. The origin of these cells, their role in cancer progression and metastasis, and possible therapeutic approaches with special implications on HNSCC are highlighted here.

## 2. Origin of Cancer Stem Cells

**Figure 1:**
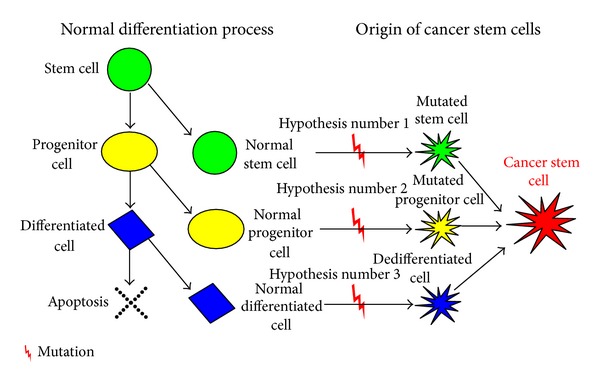
Hypothesis suggesting origin of cancer stem cells. In the process of normal differentiation a cell differentiates to form two cells, differentiated and primitive. A terminally differentiated cell is formed from precursor progenitor cell and finally undergoes apoptosis. CSC may originate from a normal stem cell (Hypothesis number 1), a normal progenitor cell (Hypothesis number 2), or a normal differentiated cell (Hypothesis number 3) by genetic mutation which will activate self-renewal genes.

Various types of stem cells give rise to progenitor cell which have the ability to further divide into specialized or differentiated cells that carry out the specific functions of the body. It is controversial as to whether CSCs arise from stem cells, progenitor cells, or differentiated cells present in adult tissue. The issue is currently under debate and the theories in origin of stem cells are presented here (Figure 1).

### 2.1. Hypothesis Number 1: Cancer Cells Arise from Stem Cells

In this scenario, cancer cells could simply utilize the existing stem cell regulatory pathways to promote their self-renewal. The ability to self-renew gives stem cells long lifespans relative to those of mature, differentiated cells [8]. It has therefore been hypothesized that the limited lifespan of a mature cell makes it less likely to live long enough to undergo the multiple mutations necessary for tumor formation and metastasis [9].

### 2.2. Hypothesis Number 2: Cancer Cells Arise from Progenitor Cells

The number of progenitor cells is more abundant in adult tissue than are stem cells. However, they retain a partial capacity for self-renewal. This property, when considered with their abundance relative to stem cells in adult tissue, forms the basis of hypothesis suggesting progenitor cells as a source of CSCs [10, 11].

### 2.3. Hypothesis Number 3: Cancer Cells Arise from Differentiated Cells

Another school of researchers have suggested that cancer cells could arise from mature, differentiated cells that somehow dedifferentiate to become more stem cell-like. In this scenario, the requisite oncogenic (cancer-causing) genetic mutations would need to drive the dedifferentiation process as well as the subsequent self-renewal of the proliferating cells. This model leaves open the possibility that a relatively large population of cells in the tissue could have tumorigenic potential; a small subset of these would actually initiate the tumor. Specific mechanisms to select which cells would dedifferentiate have not been proposed. However, if a tissue contains a sufficient population of differentiated cells, the laws of probability indicate that a small portion of them could, in principle, undergo the sequence of events necessary for de-differentiation [9]. Induction of Epithelial Mesenchymal Transition (EMT) in differentiated human epithelial cells leads to the acquisition stem cell like phenotype and formation of CSCs [12, 13]. The role of EMT in carcinomas including HNSCCs has now been well established [14].

## 3. CSCs: In Disease Progression and Metastasis

Most of the concepts in carcinogenesis and the treatment of cancer were based on hierarchical old cancer model. This traditional model, called the “clonal genetic model of cancer,” defined cancer as a proliferative disease originating from mutated tumor cells that contribute equally to the tumorigenic activity of all cancer cells within a tumor. Accordingly it has had the greatest influence on the development of existing therapeutic strategies and molecular cancer markers so far, but it does not explain some fundamental facts about tumor cells like the heterogeneity observed in a single tumor nest [15]. Also the basis of therapy on this model has not proved to be effective over the time.

A new defining model for carcinogenesis, the “cancer stem cell hypothesis,” was put forward by Wicha et al. in 2006 [16]. According to this model, cancer is a stem cell disease that places malignant stem cells at the centre of its tumorigenic activity. Stem cells at the top of their hierarchy have the capacity to undergo self-renewal and have the potential to differentiate into different types of cells in a specific lineage [17, 18]. The present model of carcinogenesis helps address most of the limitations of traditional cancer model (Table 1).

Though the acceptance of either of the model of carcinogenesis is debatable, majority of researchers now consider cancer stem cell hypothesis in defining the process of carcinogenesis. We, based on our review of various studies, consider Wicha et al. model of carcinogenesis as more significant and hence base our further discussion on this model.

The survival of any type of stem cell is largely dependent on distinctive and specific microenvironment called “niche.” The niche is the microenvironment in which stem cells reside and is responsible for the maintenance of unique stem cell properties such as self-renewal and an undifferentiated state. Niches are composed of heterogeneous populations including stem cells and surrounding differentiated cells that control critical intrinsic factors necessary in determining stem cell fate. These critical factors include stromal support cells, soluble factors, extracellular matrix proteins, and blood vessels [20]. A similar type of niche named “cancer/CSC niche” is necessary in survival of CSCs. Certain processes such as inflammation, EMT, hypoxia, and angiogenesis occur in CSC niche which helps to sustain the lethal population of CSCs [21].

Metastasis is a complex, multistep process that involves a specific sequence of events; namely, cancer cells must escape from the original tumor, migrate through the blood or lymph to a new site, adhere to the new site, move from the circulation into the local tissue, form micrometastases, develop a blood supply, and grow to form macroscopic and clinically relevant metastases [22–24]. It has been suggested that a small, and most likely specialized, subset of cancer cells drives the spread of disease to distant organs. Some researchers have proposed that these unique cells may be CSCs [8–10, 22, 25].

**Figure 2:**
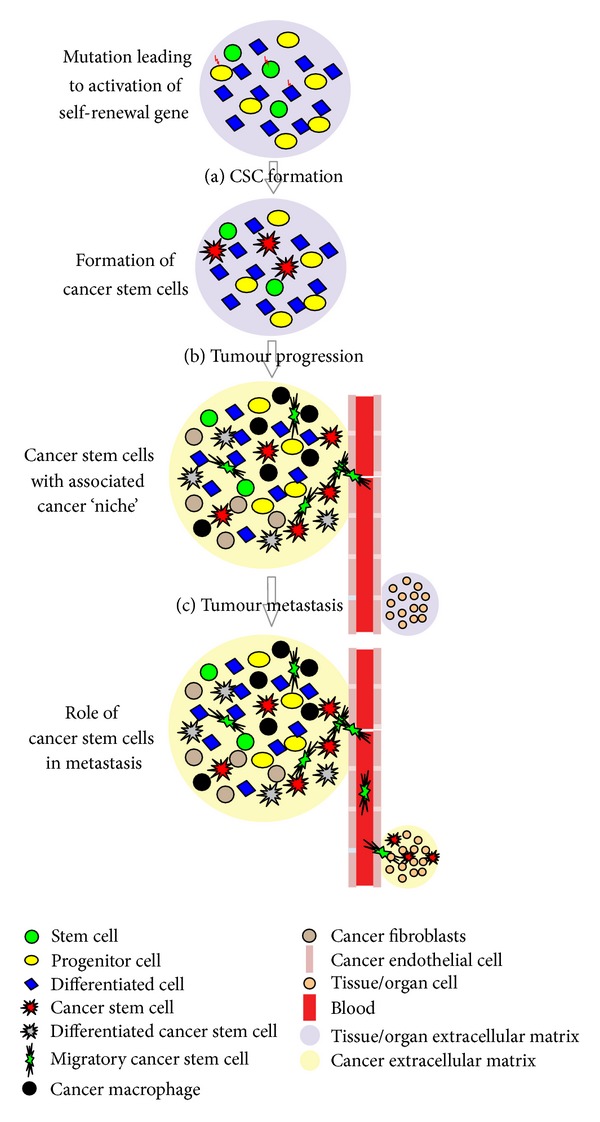
Simplified “Unifying Hypothesis” on origin of CSC, its role in tumor progression and metastasis. (a) CSC formation- mutation in normal stem, progenitor, or differentiated cell will activate self-renewal genes to form CSC. Accumulating evidence suggests the importance of “CSC niche” and its interdependence with CSCs. A “CSC Niche” will consist of plethora of molecules and cells like stem cells, surrounding differentiated cells, stromal support cells, inflammatory cells, soluble factors, extracellular matrix, and blood vessels. (b) Tumor progression—according to “Cancer Stem Cell Hypothesis” CSCs have the capacity to undergo self-renewal and have the potential to differentiate into different types of cells in a specific lineage. This accounts for heterogeneity and progression of tumor. (c) tumor metastasis—specialized stem cells described under the concept of “Migrating cancer stem (MCS)-cells” along with cancer angiogenesis play important role in tumor metastasis. MCS-cells disseminate through various portals like blood vessels to various distant organs/tissues which provide favorable environment for the growth and division of these migrated cells.

In a study based on observations made in human colorectal cancer, a concept called “migrating cancer stem (MCS)-cell” was introduced' [26]. The hallmark of this model is the existence of mobile cancer stem cells, which transiently develops from stationary cancer stem cells by combining two decisive features: stemness and EMT. Owing to the property of mobility, these MCS-cells can disseminate through various portals to favored “niche” at a distant site which supports growth and provides nutrition to these migrated cells. Changes in the microenvironment that surround these cells, such as inflammation and hormonal status, might later induce proliferation and differentiation (Mesenchymal Epithelial Transition) of disseminated MCS-cells, leading to both primary tumor recurrence and metastatic growth [26]. Recently based on various molecular studies, the concept of MCS-cell was proved to hold significance even in HNSCC [27]. A simplified “unifying hypotheses” on origin and role of CSCs in carcinogenesis is depicted schematically (Figure 2).

## 4. Cancer Stem Cells Identification

Identification of cancer stem cells based on increased expression of certain markers in cancerous tissue is the basis of target therapy which is described later in this review. By far the most common method of identifying CSCs has relied on the expression of specific cell surface antigens that enrich for cells with CSC properties. Many of these antigens were initially targeted because of their known expression on endogenous stem cells. While a multitude of studies have identified CSC markers across a variety of solid malignancies, relatively few of these markers have been studied in HNSCC. We will describe few of the proved methods by which CSCs in HNSCC can be identified.

*(1) CD133.*  A pentaspan transmembrane glycoprotein localized on cell membrane protrusions is a putative CSC marker for a number of epithelial malignancies including colorectal, brain, prostate, breast and lung [5, 28–31]. In HNSCC cell lines, CD133^high^ cells display increased clonogenicity, tumor sphere formation, and tumorigenicity in xenograft models when compared to their CD133^low^ counterparts [32, 33].

*(2) CD44. *One of the well-recognized CSC markers is a large cell surface glycoprotein that is involved in cell adhesion and migration. It is a known receptor for hyaluronic acid and interacts with other ligands such as matrix metalloproteases [34, 35]. Initially, it was identified as a solid malignancy CSC marker in breast cancer [36]. Various studies have now established the role of CD44 positive cells as a CSC marker in HNSCC [6, 37–39].

*(3) Aldehyde Dehydrogenase Activity.* The aldehyde dehydrogenase family, of which ALDH1 is a member, is a family of cytosolic isoenzymes, which are highly expressed in many stem and progenitor cells [40]. Its known functions include the conversion of retinol to retinoic acids and the oxidation of toxic aldehyde metabolites, like those formed during alcohol metabolism and with certain chemotherapeutics such as cyclophosphamide and cisplatin [41, 42]. As with CD44, the lead for investigating ALDH as a marker for CSCs in HNSCC followed identification in other solid malignancies such as breast, colon, liver, and lung tumors [43–46]. Many studies in HNSCC have proved the role of ALDH1+ cells in tumourigenesis, metastasis, and chemoresistance in HNSCC [14, 40, 47, 48].

*(4) Side Population. *Subpopulations of Hoechst 33342 dye-resistant cells termed “side population” (SP) cells have shown to express stem cell qualities when isolated from cancer samples. SP cells from OSCC have shown to be more tumourigenic and chemoresistant and have demonstrated self-renewal *in vivo*. Usually, Hoechst 33342 dye is effluxed by ATP-binding cassette (ABC) G2, so it is considered to be a CSC marker in OSCC [49]. High ABCB5 expression has shown to be associated with OSCC progression and recurrence making it a possible prognostic factor [50].

*(5) GRP78. *Recently, glucose regulated protein 78 (GRP78) was used to identify HN-CSCs from the HNSCC cell line [51]. GRP78 is an endoplasmic reticulum chaperone protein that is also expressed on the plasma membrane and is essential for survival of embryonic stem cells, presumably by acting in the ER stress response pathway [52]. GRP78 is overexpressed in several cancers including HNSCC, and coexpression of the stem cell marker Nanog with GRP78 is associated with reduced survival of HNSCC patients. GRP78 is required for tumorigenicity, invasion, and metastasis of HNSCC. Importantly, knockdown of GRP78 reduces self-renewal and tumorigenicity in nude mice suggesting that GRP78 is not merely a marker for HN-CSCs but seems to be also involved in their stemness [51].

*(6) c-Met. *c-Met, a tyrosine kinase receptor for hepatocyte growth factor (HGF), is associated with metastasis and tumor invasion, decreased survival, and was recently investigated as a marker for CSCs in HNSCC [53–55]. Clinically, conventional chemotherapy resistance involved in some types of cancers has been associated with the activated c-Met expression [54]. Hence, c-Met expression can not only be considered as a marker for CSC but also as clinically relevant therapeutic target for some patients with acquired resistance to chemotherapy. The tumorigenic potential of c-Met^+^ cells when compared to CD44^+^ cells was found to be higher. Also, the combined tumorigenic potential of c-Met^+^/CD44^+^ was found to be higher when compared to individual CSC marker [53]. Further studies with greater number of samples to determine tumorigenic potential of c-Met combined with other markers like CD44 and ALDH1 is yet to be completed.

*(7) Tumor Sphere Formation. *Under serum-free culture conditions, CSCs can be maintained in an undifferentiated state, and when driven toward proliferation by the addition of growth factors, they form clonally derived aggregates of cells termed tumor spheres [2, 56]. In HNSCC, these spheres have been shown to be enriched for stem markers, including CD44hi [57], Oct-4, Nanog, Nestin, and CD133hi [58, 59], as well as exhibiting increased tumorigenicity in orthotopic xenografts [58].

## 5. Cancer Therapy: Targeting CSCs

Besides providing a model of disease progression and metastasis, CSCs have important implications regarding cancer treatment. While current chemotherapy and radiation treatment for HNSCC are focused on indiscriminate cytoreduction, the CSC hypothesis suggests that only by eliminating CSCs can cancer be treated effectively. However, there is substantial evidence that CSCs have inherent drug and radiation resistance, rendering most conventional therapies ineffective and explaining tumor recurrence despite significant reductions in the tumor volume. The mechanism behind resistance differs. Radiation resistance is attributable to increased DNA repair, while resistance to chemotherapy is frequently related to accelerated drug transport and to drug metabolism.

The therapeutic strategies may be based on (i) targeting cancer stem cells, (ii) antiangiogenic agents, and (iii) induction of CSC differentiation and maturation. Extensive work is being done to understand the molecular mechanisms exclusive to pathobiology of CSC, which will allow specific and targeted therapy.

Targeting various signaling pathways involved in CSC formation like Notch [60], Wnt [61], and Hedgehog [62] has provided promising results in targeted CSC therapy. Many pharmaceutical companies have formulated drugs to target the pathways in CSC formation. The ability of these drugs to selectively target cancer stem cells while sparing normal stem cells remains questionable and is critical for the future application of cancer stem cell therapy. Targeting the ROS status of CSCs is also suggested to prove effective in targeted therapy by alteration of intracellular milieu which will facilitate apoptotic death signals over proliferative effects [63]. Studies to make CSCs chemoradiosensitive have been attempted in HNSCC. In HNSCC, CSCs were made more chemosensitive via knockdown of Bmi-1 and CD44 [48, 64]. Another barrier which needs to be addressed is regarding the specificity of drugs. The drugs that target CSCs must avoid damaging normal stem cells, to be clinically useful.

The niche provides the soil for CSC self-renewal and maintenance, stimulating essential signaling pathways in CSCs and leading to secretion of factors that promote angiogenesis and long-term growth of CSCs. Evidence indicating interaction of CSCs with angiogenesis in a “vascular niche” has been proved [65]. Hence, the role of targeting “vascular niche” in treatment of carcinomas cannot be neglected. This forms an important conceptual strategy for targeted elimination of cancer stem cells through the disruption of their supportive niche. In glioblastoma models, the use of antiangiogenic therapies correlated with a decrease in cancer stem cell fraction [66]. Selective elimination of tumor associated blood vessels in HNSCC xenografts using caspase-based artificial death switch (i.e., iCaspase-9) resulted in reduction of fraction of CSCs [67]. The results of antiangiogenic therapy are fascinating; however, it has to be considered with caution. It has been hypothesized that tumor cells may acquire an invasive phenotype in an attempt to escape from the unfavorable tumor microenvironment generated by the effects of antiangiogenic drugs via a phenomenon called “evasive resistance” [68].

CSC, a type of stem cell, has an inherent masked capacity of differentiation. The process is regulated by various differentiation factors like BMPs, which under normal conditions induce differentiation of neuron precursors into mature astrocytes. In mice with transplanted human brain tumor cells, BMP4 had the effect of inhibiting tumor growth. Glioma CSCs received a signal to differentiate into nonmalignant cell [69]. Similar studies based on targeting CSCs in HNSCC to differentiate into nonmalignant epithelial cells can be undertaken.

**Figure 3:**
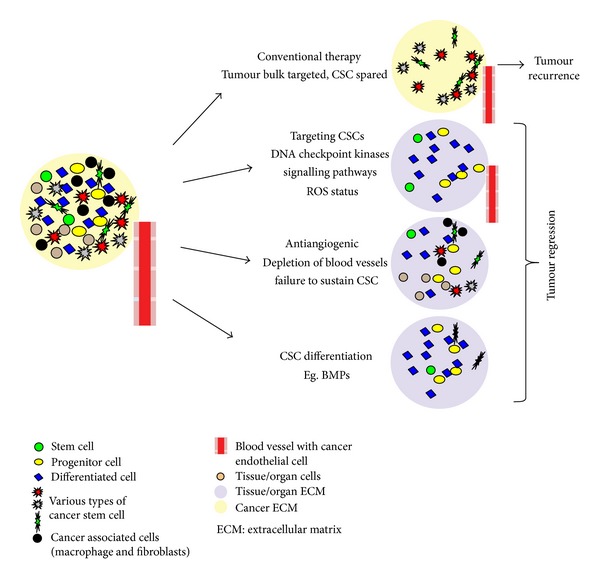
Multistrategic approach in combating cancer. Conventional therapy targets tumor bulk and CSCs are spared. The CSCs, having the potential of self-renewal and to differentiate into various cells, will lead to recurrence. Combination of conventional therapy which potentially allows for tumor debulking and “CSC targeted therapy” which may prevent recurrence and metastasis is advocated. The “CSC targeted therapy” includes (a) specifically targeting CSC by altering signaling pathways and ROS status. (b) Antiangiogenic therapy which will cause depletion of blood vessels and loss of CSC. (c) Certain differentiation factors will help in differentiating CSCs to more mature form and will lead to loss of “stemness.”

Based on the recent literature on treatment of cancer we propose a multistrategic approach which may prove effective as opposed to conventional treatment which has failed to improve the morbidity and mortality of HNSCC patients. Targeting CSCs forms the mainstay approach of the proposed multistrategic approach which combines conventional therapy which potentially allows for tumor debulking and stem cell targeted therapy which may prevent recurrence and metastasis (Figure 3).

## 6. Conclusion

Recent advances in molecular techniques have helped in better understanding the role of CSC in disease progression. Efforts and further research are still advocated to determine specific markers and methods to specifically target these cells. With the alarming rise in number of new malignancies detected worldwide and fair success rates of current therapeutic strategies, a new approach in combatting cancers which will help decreasing mortality as well as morbidity of patient needs to be urgently addressed. We are cautiously optimistic about the success of “cancer stem cell targeted therapy” which will address the shortcomings of conventional therapy and will evolve as core strategy in future of treatment of cancers. Studies pertaining to HNSCC remain limited and most of the hypothesis are based on cancers from other organs/tissues. More studies relating to HNSCC CSCs should be undertaken which will help in treatment of HNSCC. Future of CSC targeted therapy is bright and will help in making cancer treatment more successful and perhaps even curative while obviating systemic toxicity.

## Figures and Tables

**Table 1:** Important features of old and new cancer model [19].

Old cancer model	New cancer model
(i) All tumor cells are equally tumourigenic (ii) Unregulated growth is due to the accumulation of multiple mutations that promote cell proliferation with concomitant silencing of growth inhibitory genes and blunting of cell death (iii) Cancer is a proliferative disease	(i) Only a minority of cells can form new tumors (ii) Unregulated cell growth is due to a disruption in the regulatory mechanism in stem cell renewal
